# Association of alkali metal cations with phosphatidylcholine liposomal membrane surface

**DOI:** 10.1007/s00249-016-1150-1

**Published:** 2016-07-01

**Authors:** Joanna Kotyńska, Izabela Dobrzyńska, Zbigniew A. Figaszewski

**Affiliations:** 1 Institute of Chemistry, University of Bialystok, Ciolkowskiego 1K, 15-245 Bialystok, Poland; 2 Laboratory of Electrochemical Power Sources, Faculty of Chemistry, University of Warsaw, Pasteur St. 1, 02-093 Warsaw, Poland

**Keywords:** Liposomes, Phosphatidylcholine, Monovalent cations, Surface charge density, Association constants

## Abstract

Interactions of alkali metal cations (Li^+^, Na^+^, K^+^, Cs^+^) with phosphatidylcholine (PC) liposomal membranes were investigated through experimental studies and theoretical considerations. Using a microelectrophoresis technique, charge densities of experimental membrane surfaces were measured as a function of the pH of electrolyte solutions. Equilibria between the PC liposomal membranes and monovalent ions were mathematically analyzed and described quantitatively through a previously proposed theoretical model. Association constants between functional groups of PC and the studied ions were determined and used to define theoretical curves of membrane surface charge density versus pH. Theoretical and experimental data were compared to verify the model. The PC membrane was found to have the highest affinity for lithium ions, among the ions tested.

## Introduction

Biological systems involve various ions, such as halides and alkali metals. The most frequently occurring cations in living organisms are monovalent alkali metal cations of sodium (Na^+^) and potassium (K^+^) (Gagoś and Arczewska 2011). A high sodium ion concentration (100–155 mM) is observed in the extracellular media of mammalian cells, while potassium ions are present at similar concentrations inside cells (Klasczyk et al. 2010). Other alkali metal cations are of physiological importance in the biochemical processes of living organisms. For instance, depending on their concentrations, lithium (Li^+^) and cesium (Cs^+^) ions may have therapeutic or toxic effects (Aral and Vecchio-Sadus 2008). Lithium is characterized by a narrow therapeutic index. Therapeutic levels of lithium are between 0.6 and 1.2 mEq/l. Mild toxicity with lithium is usually observed at a plasma lithium level of 1.5–2.5 mEq/l and moderate toxicity at lithium levels of 2.5–3.5 mEq/l (Alexander et al. 2008). Total cesium intakes of 6 g/day have been found to produce for example severe hypokalemia, hypomagnesemia, and even acute heart arrest (Neulieb 1984). However, full knowledge of its acute and chronic toxicity is not available (Melnikov and Zanoni 2010).

Given the ubiquitous presence of monovalent ions in biological systems, studies are needed to understand their influences on biological functioning. Biological effects of metal cations on phospholipid membranes depend on the ion levels near the membrane surface and specific interactions with the phospholipid headgroups (Binder and Zschörnig 2002). Monovalent ions such as Na^+^ and K^+^ are important in modulating plasma membrane properties. Na^+^ being the major ionic species in the extracellular fluid, binds tightly to the carbonyl region of PC bilayers and change lipid packing to a significant extent, whereas K^+^, which are the main monovalent cations in the intracellular fluid, bind to PC membranes much weaker compared to sodium ions. It is connected with larger size of a K^+^, which implies a smaller ionic surface charge and a less-ordered first hydration shell (Gurtovenko and Vattulainen 2008).

Nearly 40 years ago, scientists noticed that metal ions binding to membrane surfaces is a complex phenomenon that can be described by considering chemical binding as well as electrostatic adsorption (McLaughlin 1977; Seelig et al. 1987). The strength of interactions between lipids and metal cations increases with the charge of the metal ion in the order Me^+^ < Me^2+^ < Me^3+^. However, distinct differences are noted between ions of the same charge (Akutsu and Seelig 1981). The apparent association of metal cations with lipid membranes is weaker for neutral lipids than for anionic ones, because the net negative surface charge of membranes of acidic lipids increases cation concentration near the lipid–water interface according to the Gouy–Chapman theory of the electrical double layer (McLaughlin et al. 1981). The phenomenon of ion adsorption in the electrical double layer at the membrane–solution interface is important since it can cause changes of many cell properties, both physicochemical and electrical, such as the membrane surface charge. Over the last years, a number of studies have demonstrated a significant impact of monovalent ions on lipid membranes. For example, Rappolt et al. (1998) using small- and wide-angle X-ray scattering investigated the effects of alkali chlorides on phosphatidylcholine–water bilayer systems in the Lα-phase. The authors showed that alkali chlorides can induce the formation of discretely different multilamellar lattices in PC–water systems. Cations, such as Na^+^, were shown to penetrate the phospholipid headgroup molecules, eliciting greater packing and lateral interactions of the phospholipid network (Garcia-Manyes et al. 2005).

Ion binding to lipid membrane surfaces has been explored through computational techniques, e.g., molecular dynamics simulations (Gurtovenko et al. 2005) and experimental methods, e.g., nuclear magnetic resonance (Roux and Neumann 1986; Huster et al. 2000), electrochemical impedance spectroscopy (Naumowicz and Figaszewski 2005), chronoamperometry (Naumowicz and Figaszewski 2014) and microelectrophoresis (Tatulian 1987; Huster et al. 2000; Sinn et al. 2006; Klasczyk et al. 2010). In particular, microelectrophoresis enables indirect measurements of electrophoretic mobility and membrane surface charge density values.

The present work continues the research of Figaszewski and coworkers on the adsorption of monovalent ions in aqueous solutions of alkali metal chlorides on liposomal membranes (Dobrzyńska et al. 2007; Kotyńska et al. 2008; Kotynska and Figaszewski 2014, 2015). PC was chosen for this study because it is the most widespread and dominant of the membrane phospholipids, and it is well understood and described in detail by biomembrane researchers. The choice of electrolyte solutions was dictated by the fact that many studies of lipid-monovalent ion interactions focus on the analysis of PC–Na^+^ interactions (Böckmann et al. 2003; Valley et al. 2011) whereas less data are available concerning the effects of other alkali metal cations on PC membranes (Gambu and Roux 1997). Microelectrophoresis was used to determine the dependence of the membrane surface charge density on the pH (pH range, 2–10.5) of the alkali metal chloride solutions. Quantitative characteristics of the equilibria between the PC membrane and monovalent ions are presented. The four-equilibrium mathematical model, proposed and published previously by our group (Dobrzyńska et al. 2007), was used to characterize the equilibria. Using this model, association constants of the –PO_4_^(−)^ and –N^(+)^(CH_3_)_3_ groups of PC with the studied ions were calculated. The model was validated by comparing theoretical and experimental data. In our opinion, the quantitative description of liposomal membrane properties is of great importance. A mathematical approach to the analyzed problem may significantly expand knowledge about phenomena involving biological membranes in living cells.

## Experimental

### Materials

L-α-Phosphatidylcholine from egg yolk was purchased from Sigma–Aldrich. HPLC-grade chloroform was purchased also from Sigma–Aldrich. Inorganic chemicals, i.e., LiCl, and KCl, were of analytical grade and were purchased from POCh (Gliwice, Poland). All solutions and cleaning procedures were performed with water purified using a Milli-Q system (18.2 MOhm.cm, Millipore, USA).

### Preparation of liposomes

Liposomes were prepared by a sonication method. Phosphatidylcholine was dissolved in chloroform (10 mg/ml). Then the solvent was evaporated under a gentle stream of argon to obtain dry lipid film. Then, the film was hydrated with appropriate electrolyte solution: LiCl, NaCl, KCl, CsCl (0.155 mol/l). Ultrasound disintegrator UD-20 (Techpan, Poland) with a “Sandwich” concentrator was used as an ultrasonic source. The disintegrator consists of three main parts: a power generator, an ultrasonic vibration transducer, and a sonotrode with a titanium tip. In the experiment, the tip, with a diameter 12 mm and amplitude of 16 μm, was used. The ultrasonic generator, with a maximum output power equal to 180 W, generates vibrations at a frequency of 22 kHz. Sonication was applied five times for 90 s. As heat is liberated during the process, cooling the suspension is necessary, which was carried out using an ice bath (container with a mixture of ice and dry sodium chloride). Phosphatidylcholine liposome sizes determined using a Zetasizer Nano ZS (Malvern Instruments, UK) apparatus exhibited a size distribution profile, with one population (representing approximately 90 % of all particles) with a diameter 160 nm and the other (representing about 9 % of the particles) with a diameter 30 nm.

### Microelectrophoretic mobility measurements

Electrophoretic mobilities of liposomes were determined by performing microelectrophoretic experiments on the samples and measuring the velocity of the particles using laser Doppler velocimetry (LDV) with Zetasizer Nano ZS apparatus. The measurements were carried out as a function of pH. Formed liposomes were suspended in an appropriate alkali metal chloride solution. To change the pH, the corresponding amount of acid or base was added. The reported values represent the average of at least six measurements performed at each pH value. All experiments were performed at least three times. Normally, in pH-sensitive measurements, buffers are used, for example Britton–Robinson aqueous universal buffer solutions (also used in our other studies, e.g., Petelska and Figaszewski 2002; Petelska et al. 2013). However, since universal buffers consist of mixtures of acids; there are many ions in an electrolyte solution. Therefore, the use of any buffer in our measurements would result in a large number of equilibria of which association constants we do not know and it would not be possible to achieve the aim of our studies. For this reason, during the measurements, pH was changed by addition of an acid or a base, which does not make any additional equilibria. Any significant pH fluctuations during measurements were observed (pH was stable).

From electrophoretic mobility measurements, the surface charge density was determined using Eq. (1) (Alexander and Johnson 1949).

1 $$\sigma = \frac{\eta \times u}{d},$$

where *σ* is the surface charge density, *η* is the viscosity of solution, *u* is the electrophoretic mobility, and *d* is the diffuse layer thickness.

The diffuse layer thickness was determined from the formula:

2 $$d = \sqrt {\frac{{\varepsilon \varepsilon_{0} RT}}{{2F^{2} I}}} ,$$

where *R* is the gas constant, *T* is the temperature, *F* is the Faraday constant, *I* is the ionic strength of the electrolyte, $\varepsilon \varepsilon_{0}$ is the permeability of the electric medium.

### Theory

Association constant of the equilibrium between the phospholipid in a liposome (L^−^) and the ion in volumetric solution (Me^+^):

3 $${\text{L}}^{ - } + {\text{ Me}}^{ + } \rightleftarrows {\text{LMe,}}$$

may be considered in two different ways.

In the first approach, the concentration of functional groups in liposomes refers to the unit volume, then:

4 $$K_{V} = \frac{{a_{{V - {\text{LMe}}}} }}{{a_{{V - {\text{L}}^{ - } }} \times a_{{{\text{Me}}^{ + } }} }},$$

$a_{{V - L^{ - } }}$ is the concentration of free functional groups per unit volume of solution which contains liposomes, $a_{V - LMe}$ is the concentration of functional groups associated with metal ions per unit volume of solution which contains liposomes, $a_{{{\text{Me}}^{ + } }}$ is the concentration of the metal ion per unit volume of solution.

In this approach, the concentration of (free or associated) functional groups depends on the concentration of liposomes in the solution, i.e., the concentration decreases if the solution is diluted, while the surface concentration of functional groups remains unchanged. In this case, if $a_{{{\text{Me}}^{ + } }}$ does not change—this does not affect the equilibrium, while the association constant value so defined will be changing. Then, the constant value will depend on the concentration of liposomes in the solution and thus the concentration of functional groups what disqualifies examining it as a constant, which should be independent of the concentration. Such a constant would retain requirements and nature of a constant, if functional groups would be uniformly distributed throughout volume of solution, i.e., if phosphatidylcholine would dissolve in water, which is not in the case liposomes (Petelska and Figaszewski 2000).

In the second approach, the concentration of functional groups in liposomes refers to the unit of outer surface, then:

5 $$K_{{}} = \frac{{a_{\text{LMe}} }}{{a_{{{\text{L}}^{ - } }} \times a_{{{\text{Me}}^{ + } }} }}$$

$a_{{{\text{L}}^{ - } }}$ is the concentration of free functional groups per unit of outer surface of a liposome bilayer, $a_{\text{LMe}}$ is the concentration of functional groups associated with metal ions per unit of outer surface of a liposome bilayer, $a_{{{\text{Me}}^{ + } }}$ is the concentration of the metal ion per unit volume of solution.

This manner of defining is accepted by us and consequently applied. It ensures the independence of the value so specified constant on the number of liposomes in the unit of volume. However, the number of functional groups depends on the number of liposomes per unit volume (liposome concentration). Such a way of perceiving of the problem is justified by us also that the equilibrium takes place on the bilayer surface (liposome) and does not depend on how far the nearest liposome (liposome concentration) is.

Adsorption of monovalent ions to the PC membrane surface was mathematically characterized. The four-equilibrium model was used to describe the dependence of the surface charge density of the PC liposomal membrane on the pH of the electrolyte solution. Two equilibria concerned the association of negative groups (–PO_4_^(−)^) of PC with H^+^ and Me^+^ ions (Me^+^–Li^+^, Na^+^, K^+^, Cs^+^), and two equilibria concerned the association of positive groups (–N^(+)^(CH_3_)_3_) of PC, with OH^−^ and Cl^−^ ions.

The equilibria at the PC liposomal surface is:

6 $${\text{A}}^{ - } {\text{ + H}}^{ + } \rightleftarrows {\text{AH}}$$

7 $${\text{A}}^{ - } {\text{ + Me}}^{ + } \rightleftarrows {\text{AMe}}$$

8 $${\text{B}}^{ + } {\text{ + OH}}^{ - } \rightleftarrows {\text{BOH}}$$

9 $${\text{B}}^{ + } {\text{ + Cl}}^{ - } \rightleftarrows {\text{BCl}}$$

where A^−^ is group –PO_4_^(−)^, B^+^ is group –N^(+)^(CH_3_)_3_ of PC.

The association constants are expressed in the following manner:

10 $$K_{\text{AH}} = \frac{{a_{\text{AH}} }}{{a_{{{\text{A}}^{ - } }} \times a_{{{\text{H}}^{ + } }} }}$$

11 $$K_{\text{AMe}} = \frac{{a_{\text{AMe}} }}{{a_{{{\text{A}}^{ - } }} \times a_{{{\text{Me}}^{ + } }} }}$$

12 $$K_{\text{BOH}} = \frac{{a_{\text{BOH}} }}{{a_{{{\text{B}}^{ + } }} \times a_{{{\text{OH}}^{ - } }} }}$$

13 $$K_{\text{BCl}} = \frac{{a_{\text{BCl}} }}{{a_{{{\text{B}}^{ + } }} \times a_{{{\text{Cl}}^{ - } }} }}.$$

Surface concentration of PC is given by *C*_PC_ (mol/m^2^):

14 $$a_{{{\text{A}}^{ - } }} + a_{\text{AH}} + a_{\text{AMe}} = C_{\text{PC}}$$

15 $$a_{{{\text{B}}^{ + } }} + a_{\text{BOH}} + a_{\text{BCl}} = C_{\text{PC}}$$

where $K_{\text{AH}}$, $K_{\text{AMe}}$, $K_{\text{BOH}}$, $K_{\text{BCl}}$ are association constants (m^3^/mol); $a_{\text{A - }}$, $a_{\text{AH}}$, $a_{\text{AMe}}$, $a_{{{\text{B}}^{ + } }}$, $a_{\text{BOH}}$, $a_{\text{BCl}}$ are surface concentrations of particular groups on the membrane surface (mol/m^2^), $a_{{{\text{H}}^{ + } }}$, $a_{{{\text{Me}}^{ + } }}$, $a_{{{\text{OH}}^{ - } }}$, $a_{{{\text{Cl}}^{ - } }}$ are volumetric concentrations of the solution ions (mol/m^3^), *C*_PC_ is the surface concentration of PC.

The surface concentration of PC can be determined by assuming that the surface area occupied by a single PC molecule was 65 Å^2^ per molecule (Kučerka et al. 2009, 2011).

Surface charge density of PC membrane is given by the equation:

16 $$\sigma = \, (a_{{{\text{B}}^{ + } }} - a_{{{\text{A}}^{ - } }} )F$$

where *F* is the Faraday constant.

Treating Eqs. (6–13) as a system of equations enables elimination of the following surface concentrations: $a_{\text{AH}}$, $a_{\text{AMe}}$, $a_{\text{BOH}}$, $a_{\text{BCl}}$ (from Eqs. 14, 15) and $a_{{{\text{A}}^{ - } }}$, $a_{{{\text{B}}^{ + } }}$, (from Eq. 16), the final result is the following equation describing surface charge density of PC liposomal membrane:

17 $$\frac{\sigma }{F} = \frac{{C_{\text{PC}} }}{{1 + K_{\text{BOH}} a_{{{\text{OH}}^{ - } }} + K_{\text{BCl}} a_{{{\text{Cl}}^{ - } }} }} - \frac{{C_{\text{PC}} }}{{1 + K_{\text{AH}} a_{{{\text{H}}^{ + } }} + K_{\text{AMe}} a_{{{\text{Me}}^{ + } }} }}$$

Equation (17) was simplified to a linear form at high H^+^ ($a_{{{\text{H}}^{ + } }} \to \infty$) and low H^+^ ($a_{{{\text{H}}^{ + } }} \to 0$) concentrations. As a result of the simplification of the equation, two linear equations were obtained, one correct for high hydrogen ion concentrations (Eq. 18) and one correct for low hydrogen ion concentrations (Eq. 19).

18 $$\frac{{\sigma \times a_{{{\text{H}}^{ + } }} }}{F} = \left[ {\frac{{C_{\text{PC}} }}{{1 + K_{\text{BCl}} a_{{{\text{Cl}}^{ - } }} }}} \right] \times a_{{{\text{H}}^{ + } }} - \left[ {\frac{{C_{\text{PC}} K_{\text{BOH}} K_{\text{W}} }}{{(1 + K_{\text{BCl}} a_{{{\text{Cl}}^{ - } }} )^{2} }} + \frac{{C_{\text{PC}} }}{{K_{\text{AH}} }}} \right]$$

19 $$\frac{\sigma }{{F \times a_{{{\text{H}}^{ + } }} }} = - \left[ {\frac{{C_{\text{PC}} }}{{1 + K_{\text{AMe}} a_{{{\text{Me}}^{ + } }} }}} \right] \times \frac{1}{{a_{{{\text{H}}^{ + } }} }} + \left[ {\frac{{C_{\text{PC}} }}{{K_{\text{BOH}} K_{\text{W}} }} + \frac{{C_{\text{PC}} K_{\text{AH}} }}{{\left( {1 + K_{\text{AMe}} a_{{{\text{Me}}^{ + } }} } \right)^{2} }}} \right]$$

Using linear regression the coefficients describing these linear functions can be easily obtained and then subsequently applied to calculate association constants. Knowledge of the parameters makes it possible to determine the theoretical liposome membrane surface charge from Eq. (17) and to compare it with the experimental data.

## Results and discussion

Surface charge densities of PC liposomal membranes were determined by microelectrophoresis. Electrophoretic mobility measurements were performed from pH 2 to 10.5, by using 0.155 mol/l lithium chloride (LiCl), 0.155 mol/l potassium chloride (KCl), and 0.155 mol/l cesium chloride (CsCl), as supporting electrolytes. Experimental membrane surface charge densities were calculated by using Eq. (1). Theoretical density values were calculated by the four-equilibrium theoretical model (see “Theory”).

**Fig. 1:**
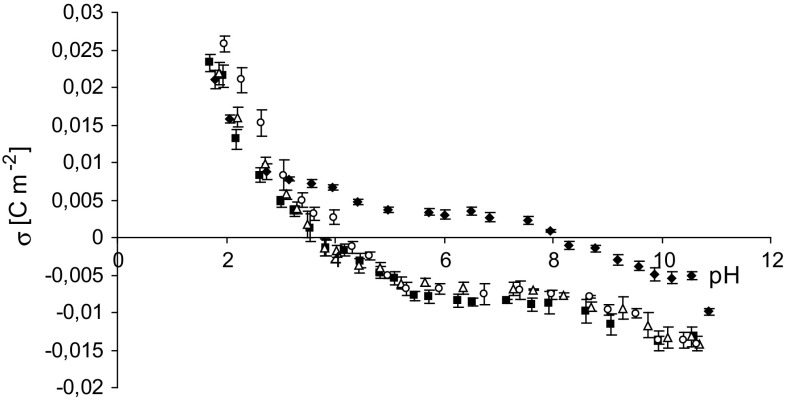
Experimental membrane surface charge densities for phosphatidylcholine liposomal membranes as a function of electrolyte pH (0.155 mol/l). *Filled diamond* LiCl, *Filled square* NaCl, *Open triangle* KCl, *Open square* CsCl

Experimental data of the surface charge densities versus pH values for the PC liposomal membrane in different salt solutions (LiCl, KCl, CsCl) are presented in Fig. 1, together with our previously obtained data using NaCl as an electrolyte (Dobrzyńska et al. 2007). Similar curves were obtained when NaCl, KCl, or CsCl was used as the electrolyte. No noticeable differences were found between the surface charge density values of the membranes (including standard deviations). Only the curve obtained with LiCl differed from the others, indicating that Li^+^ have the strongest affinity for the PC membrane. These ions caused significant changes in the surface charge density values, which transitioned from negative to positive, in the pH range from 4 to 8. The isoelectric point of the PC membrane in aqueous LiCl solution showed a significant shift towards higher pH values (pH ~8.2) compared to the membrane in the other solutions.

**Fig. 2:**
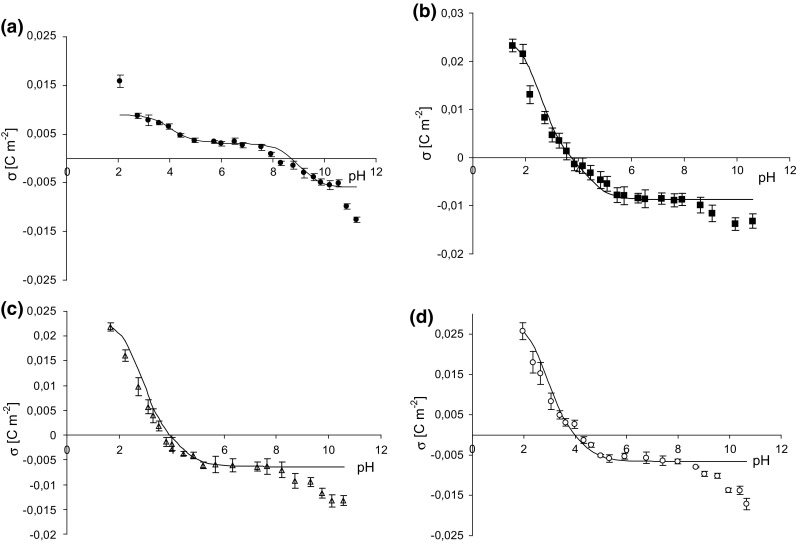
Surface charge density of phosphatidylcholine membrane versus pH of electrolyte solution. *Points* denote the experimental values,* continuous line* links the theoretical values. **a** LiCl, **b** NaCl, **c** KCl, **d** CsCl

Figure 2a–d presents the membrane surface charge densities of PC liposomal membrane in aqueous alkali metal chlorides solution plotted as a function of pH. Points denote experimental values, and continuous lines represent the theoretical values obtained from Eq. (17). A drop in the surface charge which it is observed on experimental curves at pH ~8.5 is probably related to destruction of lipid membranes at such high pH. As can be seen, there is good fitting the experimental points to the theoretical lines in pH between 2 and 8.5. Noticeable at pH > 8.5, incompatibility of experimental points and theoretical curves is due to the failure to take into account experimental points, obtained at pH > 8.5 in the theoretical model. We always evaluate a scatter in the obtained data and at such high pH measured values were characterized by a certain scatter. Since we did not trust these results, we did not use the points in association constants calculation as well as theoretical curves determination.

Parameters characterizing the liposomal surfaces are presented in Table 1. As can be seen from the table, the association constants *K*_AMe_ are of the order 0.2–0.3 m^3^/mol. In the literature, for example in the work of Klasczyk et al. (2010), the association constant for Na^+^ is equal to 1.37 l/mol. Difference in the values obtained by us and in the literature may be the result of defining the association constant. We relate concentration of solution ions to a unit of volume (mol/m^3^) while concentrations of phospholipid functional groups—to a unit of area (mol/m^2^). However, in the literature, as we understand, concentrations of phospholipid functional groups are referred to as a unit of volume. On the final dimension of the association constant unit whatever kind approach should not have affect. However, numerical values differ in association constant calculations because they are referred to as either a unit of volume (Klasczyk et al. 2010) or a unit of area (our data). Also, there is a large difference in *K*_BOH_ and *K*_BCl_ values between LiCl and other electrolytes. At this time, we are not able to give an explanation so much difference in the values. Examining the possibility of ion adsorption in the electrical double layer at the membrane–solution interface, it is thought that the influence of metal cations on the membrane surface charge increases as the cation size decreases. Lithium ions are small; the radius of Li^+^ ion is equal to 0.6 Å (Na^+^ = 0.95 Å, K^+^ = 1.33 Å, Cs^+^ = 1.69 Å) (Cotton and Wilkinson 1982); the ions also have the smallest free-solution mobility and the largest apparent hydrated size. Lithium ions are characterized by an extremely high charge density (highest of all known naturally occurring ions), which is the cause of the strongest electrostatic attraction. Therefore, among the alkali metal cations, Li^+^ have the strongest interactions with membranes. Since the interaction between alkali metal cations and functional groups of lipids has an electrostatic character, the formed systems are thus ion pairs. Lithium ions adsorb on the membrane surface, causing charge compensation (at the right pH) and even changing the charge sign. Conversely, Li^+^ show the least adsorption on metal surfaces among the alkali metal ions. The strong adsorption of Li^+^ to the PC membrane suggests that these ions at least partially (from the side of the membrane) lose their hydration shell. Our data are in agreement with previous observations of a unique influence of Li^+^ ions on the electrical properties of lipid membranes. Klasczyk et al. (2010) demonstrated that the zeta potential of PC membranes is negative in the presence of Na^+^, K^+^, and Cs^+^ (0.155 mol/l, pH 7.0) is negative, but positive in the presence of Li^+^ ion. These results indicate that the adsorption of alkali metal cations is in accordance with the Hofmeister series: Li^+^ > Na^+^ > K^+^ ~ Rb^+^~ Cs^+^. However, to our knowledge, no published study in the literature has considered this adsorption as a function of pH. The theoretical surface charge densities of PC membranes in alkali metal chloride solutions (determined by the four-equilibrium model) and the association constants of the PC functional groups with electrolyte ions confirm that the strongest adsorption is obtained with Li^+^ ions.

**Table 1:** Association constants of phosphatidylcholine functional groups with monovalent ions (Li^+^, Na^+^, K^+^, Cs^+^, Cl^−^, H^+^, OH^−^)

Electrolyte	Association constants			
	*K*_AMe_ (10^−1 ^m^3^/mol)	*K*_AH_ (10^2^ m^3^/mol)	*K*_BOH_ (m^3^/mol)	*K*_BCl_ (10^−2^ m^3^/mol)
LiCl	3.42	4.93	2.78 × 10^3^	22.2
NaCl	2.32	6.37	2.58 ×10^9^	7.35
KCl	3.33	9.12	1.52 × 10^9^	8.37
CsCl	3.24	7.31	1.09 × 10^9^	6.52

## Conclusions

The influence of the adsorption of monovalent ions on the PC membrane surface charge was determined by experimental and theoretical approaches. Among the analyzed cations, Li^+^ had the strongest affinity for the PC membrane in the experimental results. Furthermore, the membrane surface charge values obtained by the theoretical model were consistent with the experimental data.

## References

[CR1] Akutsu H, Seelig J (1981). Interaction of metal ions with phosphatidylcholine bilayer membranes. Biochemistry.

[CR2] Alexander AE, Johnson P (1949). Colloid science.

[CR3] Alexander MP, Farag YMK, Mittal BV, Rennke HG, Singh AK (2008). Lithium toxicity: a double-edged sword. Kidney Int.

[CR4] Aral H, Vecchio-Sadus A (2008). Toxicity of lithium to humans and the environment—a literature review. Ecotoxicol Environ Saf.

[CR5] Binder H, Zschörnig O (2002). The effect of metal cations on the phase behavior and hydration characteristics of phospholipid membranes. Chem Phys Lipids.

[CR6] Böckmann RA, Hac A, Heimburg T, Grubmüller H (2003). Effect of sodium chloride on a lipid bilayer. Biophys J.

[CR7] Cotton FA, Wilkinson G (1982). Advanced inorganic chemistry.

[CR8] Dobrzyńska I, Kotyńska J, Figaszewski Z (2007). Changes in electrical charge of phosphatidylcholine and phosphatidylserine liposomal membranes caused by adsorption of monovalent ions. Chem Anal.

[CR9] Gagoś M, Arczewska M (2011). Influence of K^+^ and Na^+^ ions on the aggregation processes of antibiotic Amphotericin B: electronic absorption and FTIR spectroscopic studies. J Phys Chem B.

[CR10] Gambu I, Roux B (1997). Interaction of K^+^ with a phospholipid bilayer: a molecular dynamics study. J Phys Chem B.

[CR11] Garcia-Manyes S, Oncins G, Sanz F (2005). Effect of ion-binding and chemical phospholipid structure on the nanomechanics of lipid bilayers studied by force spectroscopy. Biophys J.

[CR12] Gurtovenko AA, Vattulainen I (2008). Effect of NaCl and KCl on phosphatidylcholine and phosphatidylethanolamine lipid membranes: insight from atomic-scale simulations for understanding salt-induced effects in the plasma membrane. J Phys Chem B..

[CR13] Gurtovenko AA, Miettinen M, Karttunen M, Vattulainen I (2005). Effect of monovalent salt on cationic lipid membranes as revealed by molecular dynamics simulations. J Phys Chem B..

[CR14] Huster D, Arnold K, Gawrisch K (2000). Strength of Ca(2+) binding to retinal lipid membranes: consequences for lipid organization. Biophys J.

[CR15] Klasczyk B, Knecht V, Lipowsky R, Dimova R (2010). Interactions of alkali metal chlorides with phosphatidylcholine vesicles. Langmuir.

[CR16] Kotyńska J, Figaszewski ZA (2014). Microelectrophoretic investigation of the interactions between liposomal membranes formed from phosphatidylcholine–phosphatidylglycerol mixture and monovalent ions. Eur Phys J E.

[CR17] Kotyńska J, Figaszewski ZA (2015). Adsorption of monovalent ions to phosphatidylcholine–cardiolipin membranes. Soft Mater.

[CR18] Kotyńska J, Dobrzyńska I, Figaszewski Z (2008). Effect of monovalent ion adsorption on the electric charge of phosphatidylcholine–decylamine liposomal membranes. J Bioenerg Biomembr.

[CR19] Kučerka N, Gallova J, Uhriková D, Balgavý P, Bulacu M, Marrink SJ, Katsaras J (2009). Areas of monounsaturated diacylphosphatidylcholines. Biophys J.

[CR20] Kučerka N, Nieh M-P, Katsaras J (2011). Fluid phase lipid areas and bilayer thicknesses of commonly used phosphatidylcholines as a function of temperature. Biochim et Biophys Acta- Biomembranes.

[CR21] McLaughlin S (1977). Electrostatic potentials at membrane-solution interfaces. Curr Top Membr Transp.

[CR22] McLaughlin S, Mulrine N, Gresalfi T, Vaio G, McLaughlin A (1981). Adsorption of divalent cations to bilayer membranes containing phosphatidylserine. J Gen Physiol.

[CR23] Melnikov P, Zanoni LZ (2010). Clinical effects of cesium intake. Biol Trace Elem Res.

[CR24] Naumowicz M, Figaszewski ZA (2005). Impedance analysis of lipid domains in phosphatidylcholine bilayer membranes containing ergosterol. Biophys J.

[CR25] Naumowicz M, Figaszewski ZA (2014). Chronoamperometry insight into the pH effect on the electrical properties of bilayer lipid membrane formed from phosphatidylcholine. Int J Electrochem Sci.

[CR26] Neulieb R (1984). Effect of oral intake of cesium chloride: a single case report. Pharmacol Biochem Behav.

[CR27] Petelska AD, Figaszewski ZA (2000). Effect of pH on the interfacial tension of lipid bilayer membrane. Biophys J.

[CR28] Petelska AD, Figaszewski ZA (2002). Effect of pH on the interfacial tension of bilayer lipid membrane formed from phosphatidylcholine or phosphatidylserine. Biochim et Biophys Acta.

[CR29] Petelska AD, Naumowicz M, Figaszewski ZA (2013). The influence of pH on phosphatidylethanolamine monolayer at the air/aqueous solution interface. Cell Biochem Biophys.

[CR30] Rappolt M, Pressl K, Pabst G, Laggner P (1998). Lα-phase separation in phosphatidylcholine–water systems induced by alkali chlorides. Biochim Biophys Acta Biomembr.

[CR31] Roux M, Neumann JM (1986). Deuterium NMR study of head-group deuterated phosphatidylserine in pure and binary phospholipid bilayers. Interactions with monovalent cations Na^+^ and Li^+^. FEBS Lett.

[CR32] Seelig A, MacDonald PM, Scherer PG (1987). Phospholipid head groups as sensors of electric charge in membranes. Biochemistry.

[CR33] Sinn CG, Antonietti M, Dimova R (2006). Binding of calcium to phosphatidylcholine–phosphatidylserine membranes. Colloids Surf A Physicochem Eng Aspects.

[CR34] Tatulian SA (1987). Binding of alkaline-earth metal cations and some anions to phosphatidylcholine liposomes. Eur J Biochem.

[CR35] Valley CC, Perlmutter JD, Braun AR, Sachs JN (2011). NaCl interactions with phosphatidylcholine bilayers do not alter membrane structure but induce long-range ordering of ions and water. J Membrane Biol.

